# Why do women choose an unregulated birth worker to birth at home in Australia: a qualitative study

**DOI:** 10.1186/s12884-017-1281-0

**Published:** 2017-03-28

**Authors:** Elizabeth Christine Rigg, Virginia Schmied, Kath Peters, Hannah Grace Dahlen

**Affiliations:** School of Nursing and Midwifery, Western Sydney University, Locked Bag 1797, Penrith, NSW 2751 Australia

**Keywords:** Homebirth, Doula, Birth worker, Midwives, Regulation

## Abstract

**Background:**

In Australia the choice to birth at home is not well supported and only 0.4% of women give birth at home with a registered midwife. Recent changes to regulatory requirements for midwives have become more restrictive and there is no insurance product that covers private midwives for intrapartum care at home. Freebirth (planned birth at home with no registered health professional) with an unregulated birth worker who is not a registered midwife or doctor (e.g. Doula, ex-midwife, lay midwife etc.) appears to have increased in Australia. The aim of this study is to explore the reasons why women choose to give birth at home with an unregulated birth worker (UBW) from the perspective of women and UBWs.

**Methods:**

Nine participants (five women who had UBWs at their birth and four UBWs who had themselves used UBWs in the past for their births) were interviewed in-depth and the data analysed using thematic analysis.

**Results:**

Four themes were found: ‘A traumatising system’, ‘An inflexible system’; ‘Getting the best of both worlds’ and ‘Treated with love and respect versus the mechanical arm on the car assembly line’. Women interviewed for this study either experienced or were exposed to mainstream care, which they found traumatising. They were not able to access their preferred birth choices, which caused them to perceive the system as inflexible. They interpreted this as having no choice when choice was important to them. The motivation then became to seek alternative options of care that would more appropriately meet their needs, and help avoid repeated trauma through mainstream care.

**Conclusion:**

Women who engaged UBWs viewed them as providing the best of both worlds – this was birthing at home with a knowledgeable person who was unconstrained by rules or regulations and who respected and supported the woman’s philosophical view of birth. Women perceived UBWs as not only the best opportunity to achieve a natural birth but also as providing ‘a safety net’ in case access to emergency care was required.

## Background

Unassisted childbirth or freebirth (giving birth at home without a midwife or physician in attendance) may signify trends in maternity care practice that lead women to make this choice [1]. The choice to birth at home with a qualified midwife is not fully supported in Australia and this is demonstrated through lack of funding for this option, lack of insurance and the fact that in 2015, out of the 28,211 registered midwives who were employed in midwifery only 241 attended birth at home nationwide [2–4]. Limited numbers of women can access a homebirth choice via the State and Territory funded public hospital schemes due to restrictive eligibility guidelines, and not all States and Territories provide this service model; for example, there are only 12 services in total situated in New South Wales, South Australia, Northern Territory, Western Australia and Victoria [3]. There are very few birthing centres and access to these facilities is limited due to strict eligibility criteria, and in some States women go in to a lottery system to gain access [5]. It appears that there are a small but increasing number of women leaving the mainstream system to birth at home without medical or professional support [6]. Unassisted birth at home and birth at home with the support of an unregulated birth worker (UBW) appears to have increased and some have had adverse outcomes [6, 7]. An unregulated birth worker (UBW) can be anyone who provides support services to women during pregnancy and childbirth. They have no regulatory requirements for formal or supervised training that would enable them recognition as a registered midwife or doctor however, they may have knowledge and experience of childbirth. Unregulated birth workers include doulas; lay-midwives; childbirth educators and ex- registered midwives [7, 8]. In Australia some midwives may have lost or chosen not to remain registered and therefore become ex-registered. Legally this renders them no longer a ‘registered midwife” or eligible to call themselves a midwife. Thus, this status defaults them to be included in the category of an UBW, and indeed this is what they generally call themselves. While most UBWs do not call themselves a midwife many can continue to practice midwifery skills under the category of an ‘Unregulated Birth worker” No data exists to report the numbers of UBWs, their training or work practices in Australia.

In Australia most women give birth in a hospital (96.9%) [9]. Care is medicalised and intervention rates in labour and birth are among some of the highest in the world [10]. Concern about the low level of access to home birthing services in Australia has been highlighted by the Australian Government in its Improving Maternity Services Review (IMSR) report, 2010 [5]. This was a commonwealth funded, public consultation report conducted by the Department of Health and Aging DOHA. They received over 900 public submissions, the majority of which were from consumers. Several ‘invitation only’ round table forums with service organisations and individual’s on a range of topics were also undertaken. Collectively this formed the basis for the IMSR 2010 report. The IMSR, 2010 identified that maternity care in Australia is not meeting the needs of all Australian woman [5], and over 60% of the public submissions informing this report were from women wanting greater access to a homebirth option” [3]. Following this review however, a number of national maternity and health regulatory reforms led to regulatory, funding and insurance changes which made the choice to birth at home with privately practising midwives (PPMs) more difficult [5]. A national health practitioner’ register was established enacting legislation that required all practitioners to have Private Indemnity Insurance (PII) however, there has been no insurance for PPMs in Australia since 2001. This legislation also had provision for PPMs to acquire a service provider number that would then enable women access to the national health insurance scheme ‘Medicare’ through a rebate for their services.

A wide range of stakeholders: regulators, professional associations, colleges, insurers and consumers support the rights of women to have a choice in care provider, birth place and access to safe, high quality maternity services [11, 12]. Despite this, gaining access to a midwife who can support women during birth at home in Australia remains a challenge for some. Some women simply cannot afford to pay for a private midwife leaving them with two options: freebirth without a midwife in attendance [6] or accessing the services of a UBW to attend them during a birth at home [7, 8].

While no data exists to examine outcomes for these women in comparison to a professionally attended birth at home and hospital birth, there have been some high profile coroners’ cases reported extensively in the media [7]. One recent case in South Australia involving an ex-registered midwife resulted in a coronial inquiry into the deaths of three babies [7]. The coroner’s recommendations have resulted in the introduction of additional legislation in South Australia that has made it illegal for a UBW to assist a woman during a birth at home without a midwife or doctor present [13]. There are discussions in other Australian States and Territories about adopting this legislation. Within this context it is important to understand the perspectives of women choosing this option as well as the perspectives of UBWs. This study explored the reasons why some women choose the services of a UBW to give birth at home without a midwife in attendance.

## Methods

A thematic analysis of in depth interviews were undertaken to explore the participants’ experiences of having an UBW at their birth and also to obtain a perspective from UBWs themselves [14]. A feminist theoretical framework was used to inform and interpret the research as this facilitated deeper understandings about the factors that shape the lives of women, and in particular their needs and expectations in pregnancy and childbirth [15]. A feminist is a person whose beliefs and behaviours are based on feminism. Feminism refers to the various movements aimed at defending political, social and economic equality for women and there are several feminisms, for example liberal, social, radical [15, 16]. Feminist theory can therefore be defined as interdisciplinary, diverse and the extension of feminisms into theoretical, fictional, or philosophical discourses aimed at understanding the nature of gender inequality [15, 16]. This study was informed by “radical feminism, which argues that patriarchy is the primary cause for women’s oppression”(p.30) [15], This can be so powerful and pervasive it has become accepted as the natural order of things [15]. This approach was taken as other studies of women’s birth choices and decision-making have found that women make the choice to birth outside of the medical system to avoid patriarchal systems of power and medical management [1, 6, 17].

Ethics approval was obtained from the Western Sydney University Ethics committee. No H10281.

## Recruitment and participants

Using a convenience sampling strategy we aimed to recruit 10 participants with equal representation of women and UBWs however, only five women and four UBWs were finally recruited. All of the UBWs had also had a baby at home with an UBW in attendance and this seems to be a common pattern. Recruitment occurred using a flyer that was distributed through two consumer websites: Homebirth Australia and Maternity Choices Australia. Inclusion criteria required women to have given birth at home with a UBW and no midwife present or be an UBW who had supported women to birth at home with no midwife present, within the last 5 years in Australia. All participants who voluntarily expressed interest in the study were provided an information flyer and opportunity to ask further question before signing a consent form signalling their informed consent to participate in this study and to publish any data obtained from participants during interviews in future academic publication.

### Data collection

Each participant was interviewed once for approximately 1 h via, telephone and/or a Skype call and at a time convenient to them. This strategy respected the sensitive nature of the topic and promoted greater confidentiality whilst minimising the potential for power imbalances [18]. The interviewer (author 1) developed rapport with participants through email and text messaging enquiries prior to the interview [18]. An interview guide was developed consisting of semi-structured open-ended questioning, as this allowed flexibility to elicit greater depth of questioning and responses (Table 1.). Questioning was undertaken with sensitivity thereby providing participants adequate time to respond to questions as these strategies are known to be effective and suitable for the investigation of sensitive topics [18]. All participants were thanked for openly sharing their experiences and for their participation in this study.

**Table 1:** Interview guide for consumers (Women)

Interview guide
1. *What lead you to choose an UBW to support you with your birth at home?* a. *How did you find her?* b. *How much did her services cost?* c. *How often did you see her?* d. *What did she do for you or with you at the birth?* e. *Did she provide services post birth?* f. *How long for and what did these services entail?* g. *How did you reconcile risk vs benefit?* h. *What emergency plans did you have in place?* *i. How far away from emergency aid were you?*
*2. Why did you not choose a home birthing midwife?*
*3. How do the services of an UBW and a private midwife differ?*
*4. Can you describe your experiences of having being cared for by an unregulated birthworker at home?*
*5. What sorts of services would you like to see available to women from mainstream services?*
*6. What would you do if the services of an UBW were not available?*
*7. What are your views about the proposed legislation to prohibit UBW support women during a homebirth without a midwife?*
*8. How might mainstream services improve so you feel you have more options?*

**Table 2:** Themes

Themes	Subthemes
1. A traumatising system	
2. An inflexible system	*1. Access to midwifery model of care* *2. Inflexible models of care*
3 Getting the best of both worlds	*1. Researching options and learning to trust in self* *2. Doing it my way* *3. Having a safety net* *4. Respecting choices*
4 Treated with love and respect versus the mechanical arm on the car assembly line	*1. Respecting choices* *2. Negotiated/flexible care feels safe* *3. No obligation to conform*

## Data analysis

Thematic analysis was used to analyse the data and report patterns (themes) within the data collected. This method of analysis reports the experiences and realities of participant’s lives and how they interpret these experiences within broader social contexts while maintaining a focus on the material and other limits of reality [19]. Thematic analysis thus reflects and unravels the surface of the world of participants [19]. Interviews were digitally recorded using a QuickTime player on a computer to ensure accuracy. These were transcribed verbatim and read several times in conjunction with reflection on field notes and feminist understandings to gain not only familiarity with the data but accuracy of understanding the women’s perspectives [18]. The interviews were then thematically analysed by author 1 by labelling and coding emerging concepts initially by hand and then entered onto the software program NVivo for further coding into nodes. Nodes were printed off and read by another member of the research team (author 4) to assess emerging themes. Nodes were then combined into themes and subthemes. These subthemes were discussed, reviewed and refined following input from all the authors. The entire research team then reviewed the final analysis.

## Reflexivity

Reflexivity enables researchers to critically examine their own deep-seated views and judgements and how this may influence the research process [20, 21]. A number of years ago when the first author was working in a maternity unit, a woman who had experienced a UBW supported birth at home was transferred to hospital in a critical condition requiring resuscitation and life saving surgery. The 1^st^ author was a UK trained midwife and experienced with homebirth in the UK. She was of the opinion that an appropriately trained and registered midwife may have been a more appropriate care provider to support a woman to birth at home. However, she was also naive to the issues Australian women experienced with respect to limited forced choices and dehumanising practices. This stimulated reflection on the motivations and reasons why women might choose not to have a midwife attended birth at home. This was the catalyst for the development of the resulting research questions for this study. In acknowledging this starting point, greater awareness for the need and importance of taking a reflexive approach was highlighted. Personal reflection and questioning during analysis led to a greater depth of understanding of the research topic and for the diversity of birth experiences. Field notes were utilised during the data collection and analysis process to build self-awareness of any preconceived assumptions that may have developed as a midwife as these are known to be useful when reflexivity is inherently connected to action [21, 22]. This provided greater appreciation for the knowledge shared by participants.

## Results

All of the UBWs had themselves birthed at home with a UBW in attendance; three providers (UBW) in total were asked about their experience of giving birth as well as their experience of being an UBW. Six participants lived in a metropolitan city and three lived in rural areas. Women in metropolitan areas lived between 10 and 30 min from a hospital and their UBW. Women from rural areas lived 60 min from a hospital and between 120 and 240 min from their UBW. Of the five consumers (women), three had a low risk pregnancy one of which was a first time mother; two had a high-risk pregnancy (one of these women had a past history of a caesarean section and a post partum haemorrhage (PPH) and a severe perineal trauma. The second mother had a cholestasis during the pregnancy attended by a UBW).

Characteristics of the UBWs who had provided birthing services to participants in this study included: an ex-registered midwife, an overseas trained midwife who had never sought registration in Australia, two lay/traditional midwives and doulas (one woman reported she had two doulas present at her birth). All referrals to UBWs were informal and access to them included: word of mouth (6), at a conference (1) and on a UBW website (2). The ex-registered midwife had approximately 30 years experience as a midwife prior to becoming a UBW. The experience of the two lay midwives ranged from one to 30 years providing home birthing services to women and the doulas’ experience ranged from 6 to 8 years. UBWs charged fees ranging between $200 and $3,000 and on occasion they provided services for barter.

The analysis identified four themes: ‘A traumatising system’; ‘An inflexible system’, ‘Getting the best of both worlds’ and ‘Treated with love and respect versus the mechanical arm on the car assembly line’ (Table 2). Collectively these themes reflect the reason why the women in this study had chosen the services of a UBW to assist them to give birth at home.

## A traumatising System

The theme ‘a traumatising system’ was identified as relating to what was said to women during the provision of policy driven practices and medicalised treatments during the birth of a baby either in a hospital or at home with registered midwives. It resulted in women feeling traumatised. Central to the traumatic experience was perceptions of an impersonalised system during the very personal time of giving birth. Experiencing the system of care as traumatising motivated women to seek an alternative birthing option with a UBW. This is illustrated by the following account from one woman who had given birth in hospital.

*I had my first birth in a hospital and ended up a fourth degree tear, immediately after I had a PPH and at the same time on the table holding my baby; I was being asked to sign forms saying I was being stitched and at the same time being told I would never be able to birth vaginally again. I would have to have a caesarean and probably be incontinent for the rest of my life by bowel and bladder…I was absolutely convinced I00% I was going to die… it was so traumatic.* (Consumer 2)

One participant who had witnessed a family member giving birth in a hospital, and a friend birthing at home with hospital midwives in attendance, reported mainstream care as disempowering and disrespectful of women. She reported witnessing hospital midwives reprimanding, bullying, threatening and coercing the woman into accepting unwelcome medical interventions and treatments. This participant emphasised that birth at home cared for by a registered midwife can also be distressing. She described this as the introduction of hospital’s policies, rules and regulations into the woman’s home. The following excerpts demonstrate that in both settings, midwives were seen to be the perpetrators of coercion and disrespect.

*I was appalled at the midwives in the hospital and this was a midwifery group practice. I was just appalled…they pushed her; they scared her into having drugs. She wanted to have no drugs. The midwife just kept on saying “well if you don’t hurry up we'll be whipping you down for an emergency caesarean section so you’ve got an hour”. Putting pressure on her and just not supporting her…giving her so much pressure, forcing her to have examinations when she didn’t want them". I just wouldn’t. I was so upset with the whole operation.* (Consumer 3)

*I’ve just been at a homebirth with a homebirth midwife from the hospital and again, I found them extremely rude, they were very pushy; they told the mother off for having the baby before they got there. They wouldn’t respect the fact that she wanted to be left alone to have time with the baby. She was just saying, we need to do this; we need to do that, your baby might die if you don’t have this injection. Reprimanding her for not calling them sooner, it’s just not nice. I just wouldn't do it. (*Consumer 3)

## An inflexible system

The theme ‘an inflexible system’ related to women’s experiences of trying to find care options within mainstream maternity services that met their individual needs. Two subthemes were identified: ‘Access to midwifery models of care’ and ‘Inflexible models of care’, both of which were impacted by inflexible rules, guidelines and criteria. This collectively led women to believe the entire system of care was unappealing and inflexible.

*They are so out of their comfort zone and it’s so clinical. You can hide the clinical stuff and make it homely and you can have a double bed so the husband can stay… Birth in water is so ok yet it’s so not achievable in standard hospitals. Labouring and birthing in the shower is almost unheard of here. It’s the simplest things that are so hard to achieve… Birthing in a hospital is so not appealing on so many levels*. (Consumer 6)

*I had a lotus birth; there was no way I could do that in a hospital; they don’t not allow a natural third stage; they don’t allow you to have any natural alternative medicines or anything like that.* (Consumer 3)

### Access to midwifery models of care

Having experienced, or becoming aware of the inflexible nature of mainstream care options; women reported that despite trying to access midwifery models of care they were unsuccessful. There were several reasons for this: no availability of midwives; an inability to afford private midwives’ fees; reluctance of midwives to care for a woman at home with risk factors due to risk of being reported to the regulator, and difficulties finding a midwife who reflects the woman’s philosophical view of childbirth. The only available model of midwifery care was seen to be a medically dominated one, which followed rigid medical protocols and guidelines.

*I did make contact with the midwives down at the birthing centre but the home birthing program still had not come through and it wasn’t available… There were no other registered home birthing midwives available. It was at that period where there were no registered midwives…the publicly funded homebirth scheme was not up and running. (Consumer 4)*

*I wanted to be at home but there were no midwives around at that stage. (Consumer 6)*

*They’ve got like the syndicate, lottery system there are so many women that want to go there but there are only so many places. (Consumer 5)*

The following two women had initially approached privately practising midwives but were unable to access the services they wanted due to a lack of connection with the midwives or availability and affordability.

*With my first birth, I wasn’t very happy with the services and the people who could assist me. There wasn’t a great deal of choice. (*Consumer 7*)*

*A big part of it was not being able to afford a homebirth midwife* … *I wanted a homebirth but I couldn’t afford to have a midwife.* (Consumer 2)

The inflexibility of midwifery regulatory guidelines impacted on access, as midwives were unwilling to provide the services women requested.

*I had approached quite a number of midwives previously. A lot of the midwives wouldn’t agree to the potential of a homebirth… I found it quite hard to find someone that was willing to take the risk…A lot of midwives were talking about insurance, they didn’t want to take the risk because they didn’t want to lose their registration. (Consumer 5)*

Inflexible rules, guidelines and criteria for accessing midwifery care options were highlighted as prohibitive with women reporting they were subjected to restrictive selection criteria, The eligibility criteria for access to publicly funded homebirth schemes and birthing centres was identified as a barrier. Some women reported that caseload midwifery models of care were capped making it impossible for all women to be provided with this option. The following women did not meet eligibility criteria due to several past obstetric risk factors, namely a prior PPH, 4^th^ degree tear and miscarriages.

*I actually booked in at the birth centre. I tried to enrol in the midwifery group practice but I got knocked back with both of them because at about 12 weeks between my first two children I had a miscarriage. (Consumer 2)*

*I was out of the geographical area, I wasn’t even eligible for it…so I didn’t have access to a birthing centre, only to a hospital…In my State, the line up for the birthing centre, is ridiculous, so many women want to get into the birthing centres in this State, and can’t get in because the waiting list is too long. And so they go to the hospitals, and a lot of them have really shit births. (Consumer 7)*

One UBW described how a woman utilised her services due to the woman’s inability to satisfy selection criteria to access through mainstream care a vaginal birth after a caesarean section (VBAC).

*A VBAC mother, she just really felt she didn’t fit into the homebirth scheme because she had a caesarean and she was really determined to have the baby at home. (Provider 5)*

### Inflexible midwifery models of care

Women and UBWs mentioned midwives’ legal requirements to practice within medically determined guidelines. This included publicly funded homebirth schemes, midwifery group practices and privately practising midwives. They felt birth at home with midwives was often so governed by the medical and regulatory rules, that the midwives appeared aligned with the system and not the pregnant women. The following highlights one woman’s disappointment and sense of loss from the inflexibility of a professionally attended birth at home with a midwife after having previously experienced a UBW supported birth at home.

*She wanted to do a vaginal examination and I just thought, just leave me. I was actually annoyed with her because I had a point of reference; a different one and she annoyed me. I realised in my heart this is not necessary…It was a really fast birth my second child but she wanted to check, she got a bit nervous at some point and wanted to do a vaginal examination to check how I was progressing. I really didn’t want her to and so ok I just let her do it so she calmed down. I didn’t have that feeling with (UBW), it wasn’t intrusive at all and I really, really valued that. (Consumer 8)*

Midwives are regulated and compelled to practice according to institutional guidelines. Women viewed this as not supporting their choices and a barrier to achieving their desired birth experience. In this sense the UBW became the woman’s next best choice as a service provider.

*I felt that I would probably be pushed into having more VEs and I didn’t want any VEs or have any monitoring, I didn’t want someone entering into my conscious space. I did speak to midwives and they all basically said from a legal stand point they are required to do all that sort of thing like for their own back up pretty much, which I understand. I didn’t want to put someone in the situation where I was asking them to go against what they had to do when it was something that was making me uncomfortable and when I knew I had another option. (Consumer 2)*

Women and UBWs highlighted that prescriptive rules and medical guidelines in mainstream care frequently interrupted the birthing process and the potential for women to experience a natural birth.

*Women’s births are being interrupted unnecessarily and the potential that women have to have an empowering birth was being ignored a lot of the time; but other times, usually in the hospital setting, there was no space for it with the rules and what is happening there. (Consumer 4)*

Women viewed the medical model as over medicalised and controlling, with blanket rules applied generically to all women irrespective of their wishes, demonstrating power-imbalance and this made care inflexible and impersonal.

*You have to have a lot of tests that need to be done and you have to have an ultrasound and things with the registered midwife. I just really like it when I know the birth worker who is with me solely. It’s just me and her. There is not this higher control system that has all these rules that are a blanket rule for everyone… She doesn’t make decisions based on maybe fear of getting in trouble with the medical and legal problems and things like that.* (Consumer 5).

## Getting the best of both worlds

The theme ‘getting the best of both worlds’ had three subthemes: ‘Researching options and learning to trust in self’; ‘Doing it my way’ and ‘Having a safety net’. Having researched all care options, women concluded the only way to achieve the birth experience they wanted was to take control of it. They achieved this by finding an alternative, knowledgeable, care provider who would not interrupt the birthing process and who would stay, support and honour their wishes throughout the birth. In this sense the UBW provided women with the ‘best of both worlds’: support for her physiological paradigm of birth and birth care that made her feel safe.

*I chose her because she was extremely experienced, she was very passionate about what I wanted, she was willing to support whatever I wanted to do during the birth, that was stay at home or the be transferred or whatever… and her beliefs were inline with mine.* (Consumer 3)

*I was looking for moral support and someone to out of the intensity of the moment to just recognise we need extra help or no you’re doing fine and that is I guess a professional opinion on how things were going. (Consumer 5)*

### Researching options and learning to trust in birth

Researching options was an important subtheme that emerged from the analysis of participants’ transcripts. Women used their previous experiences with the mainstream care system as a stimulus for their own research. They researched all care options to become more knowledgeable about: birth; the long-term effects of different birth approaches both medicalised and natural; and the availability of care options to inform their decision-making for future births. They concluded that avoiding medicalised approaches to childbirth gave them the greatest potential to achieving the birth experience they wanted.

*I studied a lot of Leboyer birth and about what happens if you have a drugged birth…and what patterns you set for yourself if you have an induced birth and how that affects your life. (Consumer 3)*

*After doing a lot more reading from a variety of sources and ideologies and different perspectives and across different years of birthing, I came to appreciate that perhaps if left alone a bit more then I would perhaps get a lot further into birth, further into labour and make it a whole lot better and smoother. (Consumer 5)*

Women described how they transitioned from a position of fear about birth to trusting birth and their bodies as they researched their options.

*I moved from a fear of ‘what if’ to a bit more trusting the body and that nature would take its course…, which is very hard in a very medicalised system. I think weighing it all up having a little bit more knowledge and trust really helped me. (Consumer 5)*

*I don’t feel the need for security of knowing where the baby is or security of what the heart rate is, I don’t feel that need … you just have to trust that you know, trust it’s going to go well and trust in your abilities that it will go well. (Consumer 9)*

### Doing it my way

The subtheme ‘doing it my way’ related to women wanting to retain full control of their birth experience to ensure an intimate, comfortable and undisturbed natural birth. All of the participants communicated a deep sense of trust in their strength and abilities as women to give birth naturally. They believed birth was an instinctively natural, physiological process that every woman was capable of achieving.

*I just wanted to do my own thing, in my own way so I could really focus and be able to do what I need to do as a birthing woman without having all the intrusion… I felt that if something was going to go wrong, that I would know, I’d feel it. (Consumer 2)*

*Women know how to birth…and I trust in the natural process of birthing; women are actually capable and should not be told they can’t do it or that they are too slow…. These are the key things for me from my experience. The women can actually do it; they know how to actually give birth. (Consumer 3)*

Women reported they did not want a health professional who practised in a medical model and who may have pressured them into having unwanted interventions. One UBW believed women chose her services because they mistrusted mainstream services and rejected midwives who viewed them as part of the medical system who work to monitor, control, and manage women and their birth.

*Women don’t want a midwife that has been trained in that technocratic model. They don’t want someone who has been trained in a hospital environment to observe hospital births and to manage birth using hospital resources and hospital mentality to come into their homebirth, which is counter, intuitive. They don’t want that. (Provider 9)*

Being at home with a familiar trusted carer, surrounded by supportive family members was highlighted as a key factor to facilitating a woman to feel in control and focused on birthing their baby without interruptions.

*I didn’t particularly want that outside person coming into my home and me feeling like I had to welcome them in… I wanted that experience at home with my family, I wanted it to be intimate, I wanted it to be personal; I wanted to feel in control. (Consumer 6)*

*It was significant that she came to my house. So the visit I felt it was very much on my terms, what ever happened, I felt very much in control about everything. And I really valued that. (Consumer 8)*

### Having a safety net

While having a birth at home and retaining control of the birth experience were important to all the women in this study, none of them wanted to birth alone or unassisted. They wanted someone who was knowledgeable and experienced with birth; shared their beliefs about birth and who could stay with them to support their choices. While women reported their UBW did not claim to be a midwife, they believed their UBW had appropriate birth knowledge, skills and experience and in fact saw them very much as midwives. This belief reassured the women that the UBW could recognise, advise and help the woman by transferring her to a hospital for medical help if the UBW deemed it necessary.

*There were a few experienced doulas in the area where I live who attended free births who I trusted, but if anything was happening anything went wrong, they had the knowledge to help get me to the transport I needed… I had two doulas… I knew she had birth knowledge and backup there instead of just me and my partner being there alone. (Consumer 2)*

*I was looking for moral support and someone out of the intensity of the moment to just recognise we need extra help or no you’re doing fine. I guess a professional opinion on how things were going. I would never attempt to birth without a knowledgeable person. I wouldn’t freebirth on purpose. I think having a doula provided me a bit of security that if something would be heading in the dangerous situation, off to the hospital we go. (Consumer 5)*

All of the women in this study viewed their UBW as their ‘midwife’ and safety net, keeping watch to protect their space and providing a sense of security for them. One woman, who was also a registered midwife, described why she liked to call her UBW a midwife. She described regulated midwifery care as impersonal placing greater emphasis on the performance of medicalised treatments and often neglected the authentic human needs of a birthing woman. This is demonstrated in following statement from a consumer who was also a registered midwife.

*I still like to call her a midwife and she says “I am not a midwife”, but the discussion we had was, I like to call you a midwife or lay midwife because in every sense of the word you are more a midwife than I am a registered birth worker. You can work with the woman whereas, of course the clinical stuff is very important… often it is seen as more important that the rest of the holistic care that the midwife does …I feel you are more of a midwife than I am because the expectation to use my clinical skills outweighs my right to practice my normal midwifery, which is being with the woman. (Consumer 6)*

The women perceived having a birth worker who was not a registered midwife as a reassuring feature of UBW care. This enhanced the women’s sense of trust in birth and their ability to give birth naturally and in many ways gave them the best of both worlds.

*I loved the way she trusted birth completely and I liked the way she trusted birth so much she didn’t need to become a registered midwife… I felt very secure in her skills.. (Consumer 4)*

## Treated with love and respect versus the mechanical arm on the car assembly line

The final theme ‘Treated with love and respect versus the mechanical arm on the car assembly line’ represents the contrast of birthing inside and outside the mainstream system. It encapsulated the impact of personalised system verses an impersonalised system of care. Three subthemes were identified within this theme, which represented key components of care that impacted on women. They included: ‘Respecting choices’; ‘Negotiated/flexible care feels safe’ and ‘No obligation to conform’. Women described their care outside the system with a UBW as focused on validating the woman’s choices and position as a person and birthing woman. An important feature of this care described by women was being treated with love and respect. Women described their experience of birthing within mainstream care as impersonal, dismissive of the women as an integral part of the birthing process and focused on medical intervention and procedures that satisfied the fears of health care professionals to successfully deliver ‘a product’ with ‘the product’ being a live baby.

*In my homebirth I was treated like a person who was part of the process. The main difference for me was, at home I was treated with love and respect, like I was important; like I was part of the process. I was treated like I was a real person. Whereas in hospital, I was treated like, I was a non-person. I was invalid and unimportant, less important that the machines that were hooked up to me …In my hospital birth I was treated like an inconvenience to the point where my midwife told me to stop vocalising because I was stressing her out. I was treated like the vehicle, which produces the result and the result being the baby. The baby being what’s important, the baby being the only thing that we are there for, the baby’s the product of the process. The product is what matters. I’m the mechanical arm on the car assembly line. (Consumer 9)*

### Respecting choices

Respecting the woman’s choices was an essential feature of UBW care that women valued highly. Women reported that their UBW had been more willing than a registered midwife to respect their choices. Finding a UBW whose beliefs about birth were aligned with the woman’s beliefs generated a greater sense of trust and security that the woman’s preferences would be respected. Having this mutual understanding and deep sense of respect for the woman’s preferences and choices was critical to the development of a relationship where the woman felt in control of her birth experience and thus secure in her own decision. Enabling the woman’s wishes was paramount to the development of an empowering birth experience for women.

*I was looking for someone who wasn’t going to make me feel rushed and uncomfortable; I wanted someone who was going to respect my wishes and that was going to respect my preferences. (Consumer 3)*

*A lot of birth workers who work as doulas, work as unassisted doulas and have a lot more trust in the birthing process. They are a lot more willing to allow the woman to make her own decisions rather than taking control and doing it themselves…So it’s passing the power back to the women rather than handing her power over to the midwife or the doctor or whoever, an obstetrician. Making sure the woman is empowered to make her own decisions. She knows what is best for her and her family if she is given the right information and allowed to make her own decisions. (Consumer 2)*

### Negotiated/flexible care feels safe

Women described their relationship with the UBW as one that was on an equal social basis and this facilitated women and UBWs to work in mutually flexible ways.

*I chose her because I thought she certainly was in line with what I wanted. I thought she would support me 100% and I would be safe if I changed my mind or if something went wrong she would support me* … *It was amazing, she would do whatever so I could stay at home. She would come over at the drop of a hat, she would give me any information I wanted. She give me female companionship, (Consumer 3)*

*She was very available to everything and flexible in every way. I remember in one antenatal meeting she would talk to my partner too and say, “Look just in case things happen fast then this is what you need to do”. I found that very empowering. That approach, this is your birth; this midwife client relationship is maybe a different one; maybe the fact she is not registered midwife; it’s more on an equal social basis with her. That respect was more equal and I felt more empowered. I had more responsibility and this was reflected by the way she said, “if things go fast or if I am not there fast enough or if the shoulders get stuck you can take your finger and do this”. (Consumer 8)*

The depth of flexibility, trust, and respect was exemplified when this same woman described her moment of birth. She highlighted the unique nature of UBW care relationship and connection with the birthing woman. This UBW intuitively responded to the birthing woman’s need for nurturing by mothering the birthing woman at her most vulnerable moment by going to her in the birthing pool, holding and supporting her in her arms to transition successfully into motherhood by giving birth naturally as nature intended.

*I think some people you meet you just get a vibe. You just get that feeling that it’s something special to be in her care. I just felt I want that, I wanted to be in her care…and you know the thing with my UBW, interestingly she really empowered me and gave me lots of autonomy but in the crucial moment of birth, I didn’t want autonomy, where just you were in that moment where you feel like your first child descending and like in that moment, she did mother me, you know. Yea, I am very lucky. (Consumer 8)*

### Not that obligation to conform

Women and UBWs reported that it was significant for the UBW not to be associated with or controlled by any regulatory or medical systems of care; thus UBWs had no obligation to conform to these frameworks of service provision. This facilitated a greater sense of control and freedom to negotiate a wider range of services that were holistic, flexible and woman-centred.

*UBWs are not bound by regulations to work and the book keeping and that accountability to a higher power and all that sort of stuff. That affects our scope of what we can do…it’s just not having boxes to tick. That’s what women are looking for. (Provider 9)*

*The midwives I know that are registered, they often are talking about the frustration of the way they want to serve women to be able to fit into the registration criteria. So in that case, the UBW doesn’t have to have that controlling criteria in the way that she has to abide by. She is not controlled by that medical system. (Consumer 5)*

Having a UBW who was not part of the medical system further enabled the women to trust her abilities to birth her baby normally.

*Because she wasn’t associated with the medical system, there wasn’t that feeling that I needed medical support. There was that deep trust that this was a really natural process that was happening…I didn’t need her to have a medical degree behind her to give birth to a baby. (Consumer 4)*

It was clear from this final theme that the heart of trauma for these women was their experience of an impersonalised system of care during a very personal time of giving birth to their child.

*As in individual and on a personal level it was wonderful. She is an amazing woman and very, very supportive emotionally and caring, vary knowledgeable. She gives a lot of confidence to the birthing woman that she can do it.* (Consumer 7)

## Discussion

We explored the reasons why women choose a UBW supported birth at home from the perspective of women and UBWs. We found that most women interviewed experienced mainstream care options as limited, inflexible, disempowering, impersonal and unsupportive of women’s natural birth choices. This resulted in women feeling abandoned, disrespected and ultimately traumatised. This stimulated women to seek an alternative care option with an UBW to avoid a repeat negative birth experience within mainstream care. UBW care provided women greater flexibility, autonomy and a more personalised birth experience that resulted in the women feeling more satisfied and empowered by their UBW supported birth at home experience.

## Medicalisation of childbirth

Globally concern for increasing rates of obstetric intervention in resource rich countries [10] has resulted in some countries reversing this trend slightly [10, 23]. However, in Australia intervention rates remain high, current caesarean section rates higher than the USA (32.2%) and OECD average of (26.9%) [10]. Reasons cited for this include “reductions in the risk of caesarean delivery, malpractice liability concerns, scheduling convenience for both physicians and women and changes in physician-client relationship among others”(p.98) [10]. This raises questions about the appropriateness of some caesarean deliveries and concern given the perinatal death rate has not demonstrated a corresponding decline over the past decade [9].

Having a caesarean delivery increases a mother’s chance of death, maternal and infant morbidity and possible complications with subsequent births [10, 24, 25]. Women who experience high rates of intervention in childbirth are less satisfied with their experience [26] and the associated lack of control in decision-making. This was a significant reason cited by women in this study for choosing a UBW attended birth at home. High rates of intervention and placing significant restrictions on access to birth at home as in Australia and the USA generates higher numbers of UBW attended births [27]. Countries that have greater access to midwifery models of care and a homebirth choice have fewer UBW attended births [28, 29]. An online survey of 1063 woman conducted by Homebirth Australia in 2012 found 54% of women identified as having risk factors that would preclude them from having a publicly funded birth at home [30]. Our study findings concur with others, when women cannot access the care they want from mainstream care, they perceived inflexible with limited options and this stimulates the woman to seek alternative options outside the system [1, 6, 31].

Care that ignores or disrespects a woman and/or her birthing choices limits her ability to make informed choices and removes her sense of control [31]. The medicalisation of childbirth characteristically involves high intervention and policy driven care for example ‘Active Management of Labour’, which can be dogmatic by nature and result in impersonal protocol driven practices such as rushed and sometimes threatening, bullying and fear driven practices [31–33] rather than nurturing woman-centred supportive care practices [29]. This works to remove a woman’s sense of control in, and of, the experience which, collectively can lead to feelings of disempowerment [34]. Findings in this study are consistent with other research, which found that hospital practitioners utilise practices that can disrespect, bully and frighten women into accepting unwanted medically controlled treatments [31, 33]. Further, negative interpersonal difficulties experienced during labour and birth for example, feeling pressured, unsupported, ignored or abandoned generates a ‘hotspot’ for the development of trauma and PTSD [35]. Haraway 1991, highlighted how the patriarchal domination of scientific knowledge has served as a covert technique of social control which can dominate women not liberate them [36]. Thus society and particularly women are conditioned into believing “that ‘nature’ is our enemy and that we must control our ‘natural bodies’ “by techniques given to us by biomedical science” [36]. Women in this study experienced this as disempowering and traumatising and were motivated to avoid a repeated experience within mainstream care and thus sought an alternative birth experience with a UBW.

## Access to midwifery models of care

Maternity care in Australia is constrained within medical models of care**,** despite decades of lobbying for change [5, 37–39]. The National Maternity Services Review (MRS) 2009 stimulated a range of national maternity and regulatory reforms aimed at increasing women’s access to midwifery models of care, however, in reality this has made gaining access to these midwifery models more complex and limiting for women and midwives [8, 40, 41]. While this may in part be due to the construction of risk by patriarchal systems of care, internationally there is general agreement that all women should have the choice and access to birth at home [10, 42]. Evidence confirms that when mothers and babies receive continuity of care from a midwife throughout pregnancy, birth and the postpartum period, health benefits are significant [43]. Our findings concur with several critics have highlighted how professional practice and regulatory arrangements continue to prioritise the medical model of care [44, 45], the lack of culturally safe services [46] and a continued lack of respect for the rights of marginalised groups and women seeking birthing services at home [47].

## Inflexible system denies access

In Australia, access to midwifery models of care is dependent upon the availability of publicly funded homebirth programs and privately practising midwives being able to access private indemnity insurance (PII) to cover intrapartum care. However, PII has not been available since 2002 and not all States and Territories provide a publicly funded homebirth program [3]. Only (0.4%) of women birth at home and (2.3%) in birth centre [9]. Birthing centre care equally must abide by institutional policies and guidelines [48, 49]. Access to these models is conditional upon women meeting strict, risk criteria [3, 9]. These criteria have been criticised as rigid, inflexible, disrespectful of women and their rights to access midwifery models of care [30]. Often even if criteria are met, access to birth centre care for example, is not guaranteed due to long waiting lists and a lottery systems for access [47]. Women in this study experienced trying to gain access as onerous, anxiety inducing and inflexible. Recent Australian research has found that when women experience hospital systems and care practices as inflexible they will seek alternative care options [31]. Women in this study highlighted how their requests for some flexibility with the medical model of care, for example, physiological third stage, birth in water and a vaginal birth after a caesarean section (VBAC), resulted in coercive care practices that threatened, frightened and labelled the woman as non-compliant and in some instances denied her access to medical care. Thus, what should be a personal choice becomes subject to judgement by health professionals who insist that nature and therefore birth be controlled. Participants in this study fought against this. Trying to navigate an inflexible system that does not meet the needs of the woman can led her to value the risks of birthing at home with a UBW over not getting her needs met in the hospital system [30].

## Traumatising treatment

Trauma for the women in this study was experienced as a result of policy and procedurally driven care practices that are associated with a medically managed birth. The literature describes this as iatrogenesis or iatrogenic trauma [50]. Iatrogenesis refers to adverse effects and conditions that result from care, treatments or advice received from physicians or health care professionals [50] and this can be experienced as physical or psychological trauma [34]. Traumatic events occur when a “person experiences, witnesses or is confronted with a traumatic event or events involving actual or threaten death, serious injury or a threat to oneself or others”(p.1) [51]. In this study women experienced both physical and emotional trauma having witnessed and/or experienced medically managed birth care with a previous birth along with inflexible risk screening that prohibited their access to midwifery care options. Women perceived inflexibility as disrespectful particularly because it dismissed the women’s wishes*.* From a feminist perspective, this is interpreted as a form of oppression [16].

Trauma as a result of childbirth is well documented in the literature [31, 34, 52–55]. It can be experienced at any time throughout the childbearing journey [52]. Women who experience high levels of medical intervention and dissatisfaction with intrapartum care are at greater risk of developing a more serious condition called ‘Post Traumatic Stress Disorder (PTSD) [56]. Findings from this study are supported by other Australian research, which showed women can avoid medical models of care to prevent a repeat traumatic experience [31]. However, they like many other women who have experienced a traumatic birth did not seek a diagnosis or treatment [57, 58] and appeared to adjust well to motherhood [34]. This suggests prevalence rates for trauma may be much higher, for example, overall 24% of Australian women experience childbirth fear which is a form of trauma and this exceeds international rates of 20% [59]. This further highlights the problematic nature of defining what constitutes birth trauma, as it cannot, as some researchers suggest, simply be described as isolated to those who proceed to manifest negative psychological effects [34]. Indeed avoiding hospital birth due to past negative experiences may be a real indication of serious birth trauma.

## Regulation

The aim of regulation is to recognise and identify midwifery as a profession and to protect women and society against harm by defining the knowledge and skills necessary to work as a midwife and to enforce quality standards [60, 61]. This includes a requirement to have insurance and to work within professional rules and guidelines in collaboration with the medical profession [3, 62, 63]. While hospital midwives are covered by their intuition’s insurance, privately practising midwives have been unable to access Private Indemnity Insurance (PII) in Australia since 2002, [3]. While extensions to the timeframe by which Australian privately practicing midwives must have this exist, homebirth midwives can be unwilling to practice without it and this impacts women’s access to midwives [64]. O’Boyle, 2014, found that Irish homebirth midwives who are unable to access PII on the open market were unwilling to practice without it, but also believed that having PII did not improve their practice or guarantee good practice [64]. Midwifery “claims an ancient history of being “with women” in parturition and the authority of a broader knowledge base than that of traditional biomedicine” [65] p.6. Regulating midwifery and midwifery models of care to the control of medical guidelines and rules has generated an attitude that the professions ‘know best’ and thus can override the ethical principles of women’s choice, freedom and autonomy and the service principle of ‘being with woman’ during childbirth [65, 66]. UBW’s in this study were sympathetic to women’s cry for support and respect of their human right to choice for birth at home; they sidestepped the function of regulation and this opens up dangerous possibilities for both women and workers. Participants in this study argued that current regulation standards disrespected their choices and forced midwives to abandon women leaving them no other option but to freebirth or find allies amongst UBW’s. Key issue highlighted by the National Health Research Committed, 2010 “women have the right to decline care or advice if they choose, or to withdraw consent at any time…her choice must be respected. Importantly, women should not be abandoned because of their choice” [67] p.4.

In Australia, legislators have attempted to address the issue of UBW-attended birth at home with further regulation. Proposals are that the Health Practitioner Regulation National Law (South Australia) (Restricted Birthing Practices) Amendment Act 2013 be incorporated into the National Law Act [68] for application across all States in Australia. This South Australian (SA) legislation prohibits UBWs from attending a birth at home without the presence of either a registered midwife or doctor. The penalties for non-compliance are heavy fines and the possibility of imprisonment [69–71]. An analysis of submissions during the public consultative period for the SA legislation highlighted concerns for outlawing UBWs through further legislation and punitive measurers, as this does not address the underlying system wide issues [8] and in particular the regulatory requirement for private indemnity insurance for intrapartum care at home which is a key issue to this problem.

This analysis highlights the need for the professions, midwifery and medical, to work collaboratively to address how it does not meet women’s needs or provide safe homebirth choices. Legislators need to exercise caution with overregulating midwifery and thus impacting women’s birthing rights and restricting women’s access to registered midwives. Overregulating the profession of midwifery can produce unintended consequences of making the practice of UBW attended homebirth more covert and underground which is an even more unsafe scenario [8].

## Limitations

This was a highly sensitive topic not only for the participants but also for the authors, who were registered midwives who work in mainstream maternity care as practising midwives. While precautions were taken to limit author bias with data analysis, two authors were involved in coding and classifying themes and field note reflection. There remains the risk for author bias in this data. The sample size was small and may not be reflective of all women’s experiences of a UBW attended homebirth. This may reflect the sensitive nature of the issue and the potential for legal ramifications for providers, and possible scrutiny and persecution by the public and media. Further research is required to identify the rate to which UBWs are attending women during birth at home with no midwife present and both the positive and negative outcomes that women and their babies are experiencing. This study formed part of a larger sequential exploratory design study and has informed the development of a national survey. This survey will invite participation from women who have experienced both a positive and negative UBW attended homebirths in Australia.

## Conclusion

Recent changes to the regulations in the provision of maternity services by midwives in Australia have placed significant limitations on women’s ability to access a homebirth midwife [5, 72] The medical model of care with associated high intervention and limited choices, dominates mainstream service delivery leaving some women feeling they have limited choice in their care provider [23, 73]. Stepping outside the system to freebirth and birth at home with a UBW is increasing [6, 7]. Limited evidence exists for the reasons why women make this choice. This study sought to explore the reasons why some women choose a UBW rather than a registered midwife to attend them during a homebirth. Women in this study sought care outside the mainstream system due to dissatisfaction with the care it offered. They found the medical model narrow, restrictive, disrespectful and dismissive of their needs, whereas care outside the system with UBWs was very flexible and provided greater satisfaction and met their needs. Women placed greater value on the emotional, cultural and spiritual aspects of care to make them feel safe. While insufficient evidence exists to argue a UBW model of care produces better or worse physical outcomes, it is clear that other forms of safety (e.g. emotional) are realised within this model. All service providers need to place greater emphasis on the provision of humanised care. Legislators need to exercise caution with over regulation, which impacts women’s birthing rights and restricts access to registered midwives. The unintended consequences of over-regulating choice may be to drive homebirth even further underground*.* More research is required to establish what women and UBWs will do if legislation prohibited their practice.

## Abbreviations

AHPRA: Australian health practitioner regulatory authority
IMRS: Improving maternity services review
MRS: Maternity services review
OECD: Organisation of economic cooperation and development
PII: Private indemnity insurance
PPH: Post partum haemorrhage
PPM: Privately practising midwife
PTSD: Post traumatic stress disorder
SA: South Australia
UBW: Unregulated birth worker
USA: United States of America
VBAC: Vaginal birth after caesarean
WHO: World Health Organisation
